# ^11^C-LY2428703, a positron emission tomographic radioligand for the metabotropic glutamate receptor 1, is unsuitable for imaging in monkey and human brains

**DOI:** 10.1186/2191-219X-3-47

**Published:** 2013-06-10

**Authors:** Paolo Zanotti-Fregonara, Vanessa N Barth, Sami S Zoghbi, Jeih-San Liow, Eric Nisenbaum, Edward Siuda, Robert L Gladding, Denise Rallis-Frutos, Cheryl Morse, Johannes Tauscher, Victor W Pike, Robert B Innis

**Affiliations:** 1 Molecular Imaging Branch, National Institute of Mental Health, 10 Center Drive, Bethesda, MD 20892, USA; 2 Eli Lilly & Co., 839 S. Delaware St, Indianapolis, IN 46225, USA

**Keywords:** mGluR1, PET, Kinetic modeling, Dosimetry

## Abstract

**Background:**

A recent study from our laboratory demonstrated that ^11^C-LY2428703, a new positron emission tomographic radioligand for metabotropic glutamate receptor 1 (mGluR1), has promising *in vitro* properties and excellent *in vivo* performance for imaging rat brain. The present study evaluated ^11^C-LY2428703 for imaging mGluR1 in monkey and human brains.

**Methods:**

Rhesus monkeys were imaged at baseline and after administration of an mGluR1 blocking agent to calculate nonspecific binding, as well as after the administration of permeability glycoprotein (P-gp) and breast cancer resistance protein (BCRP) blockers to assess whether ^11^C-LY2428703 is a substrate for efflux transporters at the blood–brain barrier. Human imaging was performed at baseline in three healthy volunteers, and arterial input function was measured.

**Results:**

Overall brain uptake was low in monkeys, though slightly higher in the cerebellum, where mGluR1s are concentrated. However, the uptake was not clearly displaceable in the scans after mGluR1 blockade. Brain penetration of the ligand did not increase after P-gp and BCRP blockade. Brain uptake was similarly low in all human subjects (mean *V*_T_ with a two-tissue compartment model, 0.093 ± 0.012 mL/cm^3^) and for all regions, including the cerebellum.

**Conclusions:**

Despite promising *in vitro* and *in vivo* results in rodents, ^11^C-LY2428703 was unsuitable for imaging mGluR1s in monkey or human brain because of low brain uptake, which was likely caused by high binding to plasma proteins.

## Background

Metabotropic glutamate receptors 1 (mGluR1s) are G-coupled receptors that regulate several brain functions, including synaptic transmission and plasticity and cellular excitability, and are potentially interesting drug targets [1]. However, the development of mGluR1 radioligands for positron emission tomography (PET) has been challenging. Successful tracers should have high affinity for mGluR1 but not significantly bind to other receptors, particularly the structurally similar mGluR5. Although several potential mGluR1 PET radioligands have been described [2-8], only two [8,9] have proven successful for imaging nonhuman primates, and none have been tested in humans.

Our laboratory recently described a new ^11^C-labeled mGluR1 antagonist - ^11^C-LY2428703 - with very promising characteristics. From an *in vitro* perspective, LY2428703 has high affinity for human mGluR1s; after a competition binding assay with ^3^H-LSN456066, *K*_i_ was 2.7 ± 0.5 nM for males and 1.4 ± 0.4 nM for females. In addition, LY2428703 has high specific binding to brain homogenates displaceable only by mGluR1 antagonists and no significant affinity for other human mGluRs [10]. An *in vivo* PET imaging study of rodents expanded this favorable profile; indeed, that study found a large specific and displaceable signal in rat cerebellum, insignificant *in vivo* binding to mGluR5, and negligible accumulation of radiometabolites in brain. In addition, LY2428703 was not a substrate for efflux transporters at the blood–brain barrier, as assessed in genetic knockout mice for ABCB1 (permeability glycoprotein (P-gp)) and ABCG2 (breast cancer resistance protein (BCRP)) [10].

This study sought to determine whether ^11^C-LY2428703 could image and quantify mGluR1s in monkey and human brains in a manner similar to its demonstrated ability to do so in rodent brain. To answer this question, we intravenously injected ^11^C-LY2428703 into rhesus monkeys and healthy humans and quantified brain uptake relative to the delivery of radioligand to the brain via arterial plasma (i.e., using compartmental modeling). We also examined factors known to affect the utility of radioligands for brain imaging: blockade of entry into brain by efflux transporters at the blood–brain barrier, the density of the target (mGluR1) in brain, the affinity of ^11^C-LY2428703 for mGluR1, and binding of the radioligand to plasma proteins.

## Methods

### Radioligand preparation

^11^C-LY2428703 was synthesized as previously described [10] and according to our Investigational New Drug Application #112,494, submitted to the US Food and Drug Administration. The radioligand was obtained in high radiochemical purity (>99%) and specific activity (specific activity at injection is reported below). Labeling was done with ^11^C, because it does not modify the basic structure of the molecule.

### Saturation binding assay

P2 membrane fractions were prepared from the cerebella of male Sprague–Dawley rats (*n* = 3), male rhesus monkeys (*n* = 3), and female humans (*n* = 4). For saturation analyses, 100 μg of cerebellar membrane protein and increasing concentrations of [^3^H]LY456066 [11] were added to quadruplicate wells in the presence of binding buffer (10 mM K_2_HPO_4_, 1.2 mM KH_2_PO_4_, 100 mM KCl, 0.1% DMSO, 0.05% ethanol and 0.1% BSA, pH 7.6). Nonspecific binding was defined by heterologous displacement of 1 μM LY480084 (mGlu1 antagonist) in select wells. Plates were incubated on ice for 75 min and membranes harvested on 0.3% polyethylenimine pretreated Wallac filter mats using a Tomtec Harvester MachII (Tomtec, Hamden, CT, USA). Dissociation constant (*K*_D_) and receptor density (*B*_max_) values were obtained in GraphPad Prism using standard nonlinear one-site binding equations. Data are presented as mean ± SEM (*n* = 3 to 4); one-way analysis of variance followed by Tukey's *post hoc* test.

### Radiochromatographic analyses

Plasma analyses were performed on a reverse-phase chromatography C_18_ column (Novapak, 4 μm, 100 × 8 mm, Waters Corp. Milford, MA, USA) using Radial-Pak® compression module RCM-100 with a sentry pre-column. Human plasma analyses were performed with a mobile phase of MeOH/H_2_O/Et_3_N, 65:15:0.1 by volume, at a flow rate of 2.5 mL/min; nonhuman primate plasma samples were analyzed with a mobile phase of MeOH/H_2_O/Et_3_N, 72.5:27.5:0.1 by volume, at a flow rate of 1.5 mL/min. To compare the outcome from both systems, elution volume is reported rather than retention time.

#### In vitro stability of ^11^C-LY2428703 in human and nonhuman primate blood

Nonradioactive anticoagulated blood was obtained on the day of study from either human or nonhuman primates. Aliquots of ^11^C-LY2428703 (about 150 kBq) were added to 0.5 mL of whole blood or 0.5 mL of plasma. Samples were incubated at room temperature for 30 min, after which either 450 μL of the radioactive plasma was added to 720 μL of acetonitrile or 200 μL of the radioactive whole blood was added to 300 μL of water. The tube containing the blood suspension was shaken well to mix and lyse the blood cells. Seven hundred and twenty microliters of acetonitrile was subsequently added and mixed well to precipitate the proteins, followed by 100 μL of water, which was again re-mixed. Radioactivity in the tubes was measured in a gamma counter and then centrifuged at 10,000×*g* for 1 min. The clear supernatant liquid was injected into the high-performance liquid chromatography (HPLC) column and the precipitate counted to calculate the recovery of radioactivity into the acetonitrile.

The stability of the radioligand in whole blood or plasma was calculated as the ratio of the HPLC % composition of samples divided by the radiochemical purity of the radioligand.

#### Radiometabolites of ^11^C-LY2428703 in human and nonhuman primate plasma in vivo

Arterial plasma was analyzed as previously described [12]. Briefly, after ^11^C-LY2428703 injection into either human or nonhuman primate, the input function was determined by periodically drawing anticoagulated blood samples. Blood aliquots were removed so that the γ-counter could provide information necessary for vascular correction. The remaining blood was then centrifuged, and plasma (450 μL) aliquots were removed and placed in acetonitriles (720 μL/ea) spiked with carrier LY2428703. The samples were mixed well, 100 μL water aliquots were added, and then, the sample was further mixed. The samples were counted in the γ-counter and centrifuged at 10,000×*g* for 1 min. The clear supernatant liquids were injected onto the radio-HPLC as described above. The precipitates were then counted for radioactivity to allow the calculation of the efficiency of recovery of radioactivity in the acetonitrile. Human plasma samples were separated with a mobile phase of higher polarity (MeOH/H_2_O/Et_3_N, 65:15:0.1 by volume) to ensure that the parent radiochromatographic peak comprised a single radiochemical species.

#### Plasma free fraction

The plasma free fraction (*f*_p_) of ^11^C-LY2428703 was measured by ultrafiltration through membrane filters (Centrifree; Millipore, Billerica, MA, USA) as previously described [13]. Briefly, about 740 kBq of ^11^C-LY2428703 (approximately 5 μL) was added to 650 μL of plasma. The mixture was incubated at room temperature for 10 min and then processed as described previously [13]. When radioactivities for the filter components were high, they were allowed to decay until they were within the optimal range of the γ-counter. Determining the free fraction during preblocking was done by drawing blood samples after the preblocking treatments were initiated but immediately before administering the radioligand.

### Monkey studies

Four male rhesus monkeys (*Macaca mulatta*, 11.7 ± 2.9 kg) were imaged in 13 PET scans. Seven scans were performed at baseline, three scans were performed after receptor blocking to calculate nondisplaceable uptake (*V*_ND_), and three scans were performed after blockade of the efflux transporters at the blood–brain barrier. Receptors were blocked by mixing ^11^C-LY2428703 with an mGluR1 antagonist - either nonradioactive LY2428703 (2.0 mg/kg) or LY2332084 (0.5 mg/kg) - in the same syringe. Efflux transporters were blocked in two scans by injecting DCPQ, which selectively blocks the efflux transporter ABCB1 (P-gp), and in one scan by injecting elacridar, which blocks both ABCB1 (P-gp) and ABCG2 (BCRP).

All studies were conducted in accordance with the National Institutes of Health *Guide for Care and Use of Laboratory Animals.*

#### PET scans

PET images were acquired using either the high-resolution research tomograph (HRRT) or the FOCUS 220 scanner (Siemens/CPS, Knoxville, TN, USA) for 120 min in 33 frames, with frame durations ranging from 30 s to 5 min, and correcting for attenuation and scatter. About 2 h before the PET scan, anesthesia was initiated with ketamine (10 mg/kg IM) and then maintained with 1% to 3% isoflurane. Electrocardiograph, body temperature, heart rate, and respiration rate were measured throughout the experiment. At baseline, the injected activity was 210 ± 51 MBq (specific activity at time of injection 43.2 ± 25.6 GBq/μmol, mass dose 6.9 ± 4.7 nmol or 0.62 ± 0.35 nmol/kg). Tissue time-activity curves were obtained from semi-automatic regions of interest encompassing the whole cerebellum and the rest of the brain. Brain uptake was expressed as standardized uptake value (SUV). Arterial input function was obtained for eight scans: four at baseline, two after blocking with nonradioactive LY2428703, one after blocking with DCPQ, and one after blocking with elacridar. Blood samples (0.5 mL each) were drawn through a port connected to the femoral artery at 15-s intervals until 2 min, followed by 1-mL samples at 3, 5, 10, 30, 60, 90, and 120 min. The plasma time-activity curve was corrected for the fraction of unchanged radioligand following the procedure detailed above.

To assess the effects of blocking agents on the *f*_P_ of ^11^C-LY2428703, *f*_P_ was measured in three monkeys both at baseline and after blockade. The first monkey was blocked with cold LY2428703 and *f*_P_ was measured 2.5 min after blocker injection (which corresponds to the maximal concentration in plasma of LY2428703), and again at 40 min. The other two monkeys were blocked with DCPQ and elacridar, respectively.

### Whole-body biodistribution and radiation dosimetry

Two male rhesus monkeys (14.1 and 19.9 kg) were scanned after intravenous injection of 318 and 274 MBq of ^11^C-LY2428703, respectively. Dynamic two-dimensional scans were acquired on the GE Advance tomograph (GE Medical Systems, Milwaukee, WI, USA) in five bed positions of the body (head to upper thigh), with frames of increasing duration (15 s to 4 min) for a total scan time of up to 115 min.

Images were analyzed by placing regions of interest on the dynamic tomographic images. Regions were drawn in identifiable source organs: brain, heart, lungs, liver, spleen, gallbladder, kidneys, lumbar vertebrae, small intestine, testes, and urinary bladder. Because the images did not include the body below mid-thigh, organ uptake was corrected for this recovery. The mean recovery of activity in the body above the thigh measured by PET was 85.6% of the injected activity measured by a dose calibrator. This recovered total activity in the body was used as the new injected activity for each scan.

At each time point, the activity measured within the organs was converted into the fraction of the injected dose by dividing the organ activities by the recovered injected activity. The area under the curve (AUC) of each organ was calculated by the trapezoidal method until acquisition ended. The area under the curve after the acquisition of the last image (i.e., to infinity) was calculated by assuming that the decline in radioactivity after this time point occurred only via physical decay, without any further biological clearance.

Residence times from the monkeys were converted into corresponding human values by multiplying with a factor to scale organ and body weights: (*b*_m_/*o*_m_) × (*o*_h_/*b*_h_), where *b*_m_ and *b*_h_ are the body weights of monkey and human, respectively, and *o*_m_ and *o*_h_ are the organ weights of monkey and human, respectively.

Radiation absorbed doses were calculated from the residence times for each source organ. We used the model for a 70-kg adult male in the OLINDA/EXM computer program [14].

### Human studies

Three healthy females participated in the study (29 ± 2-year old, 69 ± 13 kg). All were free from current medical and psychiatric illnesses, as assessed by medical history, physical examination, electrocardiogram, urinalysis including drug screening, and blood tests (complete blood count, serum chemistries, thyroid function test, and antibody screening for syphilis, HIV, and hepatitis B). The National Institutes of Health Central Nervous System Institutional Review Board approved the protocols and the consent forms. Written informed consent was obtained from all subjects.

#### PET scans

PET images were acquired using the GE Advance scanner (GE Healthcare, Milwaukee, WI, USA) for 120 min. An 8-min ^68^Ge transmission scan was obtained before the injection of the radiotracer for attenuation correction. The mean injected activity was 328 ± 12 MBq. The specific activity at the time of injection was 87.2 ± 34.7 GBq/μmol, which corresponded to 5.5 ± 2.3 nmol (0.082 ± 0.037 nmol/kg) of carrier. Blood samples (1 mL each) were drawn from the radial artery at 15-s intervals until 150 s, followed by 3-mL samples at 3, 4, 6, 8, 10, 15, 20, 30, 40, and 50 min, and 4.5 mL at 60, 75, 90, and 120 min. The unchanged parent fraction in plasma and *f*_P_ of ^11^C-LY2428703 were determined as described above.

#### Magnetic resonance imaging

To identify brain regions, magnetic resonance (MR) images were obtained using a 3-T GE Signa device (GE Healthcare, Milwaukee, WI, USA). T1-weighted structural images were acquired with a voxel size of 0.86 mm × 0.86 mm × 1.2 mm. The image acquisition sequences were the time of repetition (7.3 ms), echo time length (2.8 ms), and flip angle (6°).

#### Image analysis

The average PET image created from all frames was first coregistered to the individual MR image. Then, both MR and all PET images were spatially normalized to a standard anatomic orientation (Montreal Neurological Institute (MNI) space) based on transformation parameters from the MR images. A volume-of-interest template [15] as implemented in PMOD (PMOD Technologies Ltd, Zurich, Switzerland) was used to obtain brain time-activity curves.

### Kinetic analysis

Volumes of distribution (*V*_T_) were obtained by nonlinear compartmental analysis. Goodness-of-fit by nonlinear least squares analysis was evaluated using the Akaike Information Criterion (AIC) and Model Selection Criterion (MSC). The most appropriate model is that with the smallest AIC and the largest MSC score. Goodness-of-fit by the compartment models was compared with F statistics [16]. A value of *P* < 0.05 was considered significant for F statistics. The identifiability of kinetic variables was calculated as standard error obtained from the diagonal of the covariance matrix [17] and expressed as a percentage of the rate constant.

## Results

### Saturation binding assay

Human cerebellum had a *B*_max_ value (186 nM) higher than that of the cerebellum of rats (167 nM) and monkeys (120 nM). However, humans also had a higher *K*_D_ value (11.1 nM) compared to rats and monkeys (6.6 nM for both species). Therefore, humans displayed a *B*_max_/*K*_D_ ratio (16.9) lower than that of rats (25.3) but similar to that of monkeys (18.2) (Table 1).

**Table 1:** ***In vitro *results (*B***_**max **_**and *K***_**D **_**in the cerebellar tissue and plasma free fraction) for rats, monkeys, and humans**

	***B***_**max **_**(nM)**	***K***_**D **_**(nM)**	***B***_**max**_**/*K***_**D**_	***f***_**p **_**(%)**	***f***_**p **_**× *B***_**max**_**/*K***_**D **_**(=*V***_**S**_**)**
Rats	167 ± 24 (3)	6.6 ± 0.3 (3)	25.3	2.7^a^	0.683
Monkeys	120 ± 8.6 (3)	6.6 ± 0.5 (3)	18.2	0.884	0.161
Humans	186 ± 11.6 (4)*	11.0 ± 1.6 (4)	16.9	0.094	0.016

*n* in parentheses. *p < 0.05 monkey vs. human. ^a^Taken from [10].

### Monkey studies

#### Pharmacological effects

^11^C-LY2428703 injection caused no significant changes in pulse, respiratory rate, or electrocardiogram.

#### Plasma analysis

The radioligand was completely stable at room temperature in monkey whole blood and plasma. After intravenous injection, the concentration of ^11^C-LY2428703 peaked at 1 to 1.5 min and then declined following a curve that was well fit with a tri-exponential function. At baseline, the parent became 50% of the total plasma radioactivity between 7 and 60 min (*n* = 4) after injection. Two radiometabolites, less lipophilic than ^11^C-LY2428703, were detected with retention volume at 3.2 ± 0.7 mL and 5.2 ± 1.7 mL while the parent's retention volume was 7.2 ± 1.6 mL (*n* = 120). The average *f*_*p*_ value from three baseline monkeys was 0.884 ± 0.37%.

#### Brain images

Brain uptake was low, but the cerebellum (the region with the highest density of mGluR1s) had a visibly, though slightly, greater uptake than the rest of the brain. The peak uptake in the cerebellum was 3.1 ± 1.4 SUV at approximately 2 min and decreased to 50% of that value at about 20 min. The peak uptake in cerebellum was about 50% higher than that in forebrain (2.2 ± 0.8 SUV at approximately 2 min). However, after mGluR1 blockade, peak uptake increased, rather than decreased, both in cerebellum (4.9 ± 0.8 SUV) and in forebrain (3.6 ± 1.9 SUV). This increase was not explained by an increase in the arterial plasma input functions or by an increase of ƒ_p_. In fact, in the monkey that underwent an mGluR1 blocked scan with nonradioactive LY2428703, *f*_*p*_ values were lower after blockade (1.12 ± 0.18%, *n* = 4, at 2.5 min; and 0.89 ± 0.14%, *n* = 4, at 40 min) than at baseline (1.25 ± 0.12%, *n* = 4).

#### Kinetic analysis

Although the receptor blocking studies showed no displaceable (i.e., no specific) binding based on peak uptake, the images were more accurately quantified using the entire scan duration and correcting for delivery of radioligand to brain, namely, compartmental modeling. Nevertheless, using a two-tissue compartment model (which showed a better goodness-of-fit than one-compartmental model), we found no evidence of specific binding based on the blockade studies. The mean *V*_T_ value in the baseline scans was 3.5 ± 1.0 mL/cm^3^, but *V*_ND_ calculated from mGluR1 blocking studies was unexpectedly higher than the baseline value (4.4 ± 2.9 mL/cm^3^).

Low brain uptake of ^11^C-LY2428703 was not caused by the radioligand being a substrate at the blood brain barrier for the two most prevalent ABC transporters: P-gp and BCRP. In fact, blocking the blood–brain barrier efflux transporters did not increase brain uptake. For DCPQ, which selectively blocks P-gp, the *V*_T_ in cerebellum was 4.4 mL/cm^3^ at baseline and 4.2 mL/cm^3^ after blockade. For elacridar, which blocks both P-gp and BCRP, *V*_T_ in cerebellum was 2.0 mL/cm^3^ at baseline and decreased to 0.8 mL/cm^3^ after blockade. Pharmacologic doses of these two blocking agents might have displaced radioligand bound to plasma proteins, thus indirectly increasing brain uptake, but such was not the case. In the DCPQ blocking experiments, *f*_*p*_ was similar at baseline (1.074% ± 0.15%, *n* = 3) and after blockade (1.100% ± 0.04%, *n* = 3) scans. For the elacridar blocking experiment, where *V*_T_ decreased after blocking, *f*_*p*_ actually doubled from baseline (0.456% ± 0.05%, *n* = 3) to post-blockade (0.912% ± 0.05%, *n* = 3).

### Whole-body biodistribution and radiation dosimetry

Intravenous ^11^C-LY2428703 injection caused no significant changes in electrocardiogram, heart, or respiration rates from baseline values. ^11^C-LY2428703 appeared to be excreted through both urine and bile in monkey, as there was activity in the urinary bladder and transient bile in the small intestine.

The liver had the highest uptake of ^11^C-LY2428703, with an average peak of approximately 12 SUV injected activity at about 5 min post-injection. Activity in the gallbladder progressively increased throughout the scan. The other organs exhibited a lower uptake of radioactivity, generally between 2 and 5 SUV. Radiation-absorbed doses were highest in the testes (13.7 μSv/MBq), the liver (9.9 μSv/MBq), and the heart wall (8.2 μSv/MBq). Lower doses (always less than 7 μSv/MBq) were estimated for the other organs (data not shown). The final effective dose was 5.8 μSv/MBq.

### Human studies

#### Pharmacological effects

Injection of ^11^C-LY2428703 caused no pharmacological effects as assessed by vital signs, electrocardiogram, and laboratory testing as well as verbal reports from all three subjects.

#### Plasma analysis

The radioligand was quite stable *in vitro* in whole blood at room temperature for 30 min. Tracer stability was 99.5 ± 1.1% (*n* = 3) in whole blood and 99.4 ± 1.4% (*n* = 3) in plasma. *In vivo*, only traces of radiometabolites were detected in plasma during the entire 2-h period of the imaging study so that the parent radioligand remained close to 99% total blood radioactivity. Human plasma samples were analyzed under chromatographic conditions where the ^11^C-LY2428703 was retained on the column for an even longer period of time than for monkey plasma. This was done to ensure efficient separation from any potential plasma radiometabolites. This does not necessarily mean that ^11^C-LY2428703 was not metabolized but rather that the fate of the radiometabolites is unknown. For example, the absence of radiometabolites from human plasma might be due to organ entrapment. The concentration of ^11^C-LY2428703 peaked at 1.5 ± 0 min (33 ± 5 SUV) and then slowly declined following a curve that was well fit as a tri-exponential function. The parent radioactivity eluted at 6.6 ± 1.5 mL (*n* = 64). The ƒ_p_ levels were 0.094 ± 0.01% (*n* = 3).

#### Brain images

**Figure 1:**
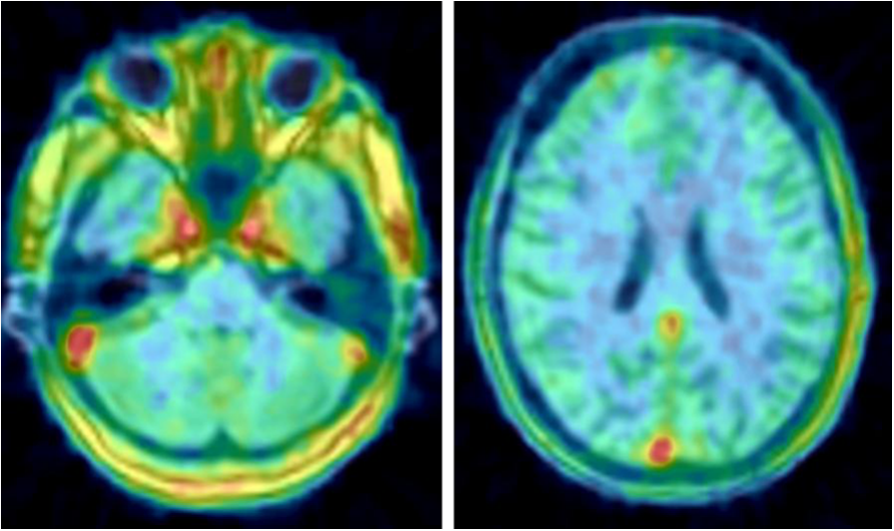
**Transverse brain slices from a healthy volunteer at the level of the cerebellum (left) and frontal cortex (right).** Images are the sum of all PET frames coregistered to the individual MRI. The foci of high ^11^C-LY2428703 uptake were due to vascular activity (carotids and posterior venous sinuses) at early time points. Vascular activity was clearly visible on the final summed image because uptake in the brain regions was negligible.

**Figure 2:**
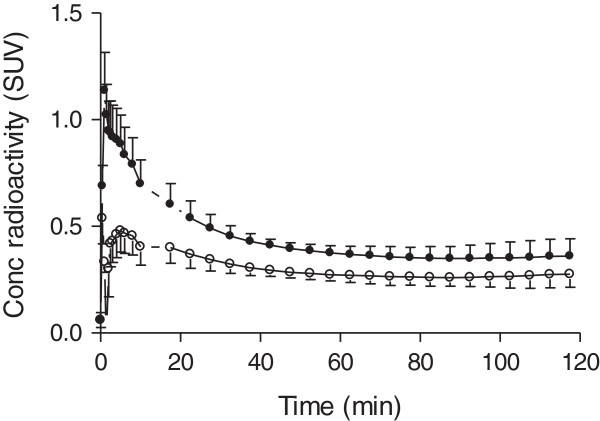
**Whole-brain time-activity curves without (black-filled circle, ●) and with (white-filled circle, ○) vascular correction.** The measured whole blood radioactivity was used for vascular correction, assuming that blood constitutes 5% brain volume.

After ^11^C-LY2428703 injection, the brain was poorly visualized in all subjects (Figure 1), with a peak uptake of 1.14 ± 0.18 SUV that occurred at 1.3 ± 0 min after injection, followed by a gradual wash-out. Notably, the brain peak uptake occurred before the arterial peak in the radial artery, suggesting that the peak in the brain was mostly due to vascular activity. Indeed, when the brain time-activity curves were corrected for the vascular component, the peak disappeared (Figure 2).

Brain activity decreased to 50% of the peak after an average of 20 min. Although a slightly higher uptake in the cerebellar cortex was observed (Figure 1), the uptake was fairly homogeneous throughout the brain. All brain activity was likely due to the parent compound, as only negligible amount of radiometabolites were detected in plasma (approximately 1% at 2 h, see above).

#### Kinetic analysis

Although brain uptake was low, we wondered whether kinetic analysis of the brain and plasma data would suggest any specific binding in the brain. In fact, *V*_T_ values with 2TCM were very low (<0.1 mL/cm^3^) and were similar across all brain regions, including the cerebellum (Table 2).

**Table 2:** Kinetic modeling results in humans using a two-tissue compartment model

	**Compartmental distribution volume**
Region	*V*_T_ (mL · cm^−3^)
Frontal cortex	0.092 ± 0.016 (14.2%)
Temporal cortex	0.102 ± 0.010 (4.4%)
Parietal cortex	0.087 ± 0.011 (4.9%)
Caudate	0.086 ± 0.024 (22.6%)
Cerebellum	0.090 ± 0.010 (2.8%)

Representative brain regions from the three human subjects. Values are mean ± SD. Standard errors are listed in parentheses and are expressed as percent (%) of the variable itself.

## Discussion

Our laboratory recently described ^11^C-LY2428703, a new mGluR1 antagonist with promising characteristics based on *in vitro* testing and on *in vivo* PET imaging of rodents [10]. However, the present study found that ^11^C-LY2428703 cannot image or quantify mGluR1s in monkey or human brain because of very low brain uptake, largely caused by high binding of the radioligand to plasma proteins.

In monkeys, the overall uptake was quite low, though slightly higher in the cerebellum, where mGluR1s are concentrated. Nevertheless, even in the cerebellum, the uptake was not clearly blocked by pharmacological doses of an mGluR1 antagonist.

Despite these poor results in monkeys, we decided to test the tracer in humans for two reasons. First, our *in vitro* analyses of cerebellar tissues showed that humans had higher *B*_max_ values (186 ± 11.6 nM) than monkeys (120 ± 8.6 nM). Therefore, ^11^C-LY2428703 could have provided a larger signal in humans. Second, results in monkeys do not always approximate those in humans. For example, ^18^F-SP203, a radioligand for mGluR5, displayed problematic characteristics in both rodents and monkeys due to defluorination in the brain and/or periphery [18]. Nevertheless, defluorination was relatively low in humans, and brain distribution volume could be robustly calculated with compartmental modeling [19]. In the present study, however, ^11^C-LY2428703 had low brain uptake in both humans and monkeys.

These disappointing results in human and nonhuman primates were unexpected in light of the high *in vitro* affinity of this ligand for human and monkey receptors and the promising *in vivo* results in rats and mice [10]. Several factors may have contributed to these results: (1) efflux transporters at the blood–brain barrier, (2) low receptor density in humans, (3) lower *in vivo* binding affinity compared to that measured *in vitro*, (4) low delivery of the radioligand to brain, and (5) low binding potential in the brain. As discussed in greater detail below, we think that the last two factors were the most problematic.

Specifically, low brain penetration may have been due to the activity of efflux transporters at the blood–brain barrier. Whether ^11^C-LY2428703 is a substrate for these transporters in humans is unknown. However, our previous *in vitro* analyses found that ^11^C-LY2428703 was not a substrate for P-gp; *in vivo* imaging studies conducted in knockout mice further found that ^11^C-LY2428703 was not a substrate for either P-gp or BCRP [10]. In addition, the current study showed that ^11^C-LY2428703 was not a substrate for P-gp or BCRP in monkey based on pharmacological blockade.

Another possible explanation is the differing receptor density between rodents and primates. However, in the present study, cerebellar *B*_max_ values were slightly higher in humans (186 ± 11.6) than in rats (167 ± 24) and monkeys (120 ± 8.6) (Table 1). Other published studies similarly found higher *B*_max_ for humans (82 ± 33 nM) than for monkeys (53 ± 12 nM), although values for rats were much higher (430.2 ± 204.2 nM) [8,20,21]. Moreover, species differences may be responsible for alterations in the structure of the receptor. However, this is unlikely to be the reason for the poor imaging properties of ^11^C-LY2428703 in primates. In fact, although *in vitro K*_D_ values are higher in humans (11.0 ± 1.6 nM), they are very similar between monkeys (6.6 ± 0.5 nM) and rats (6.6 ± 0.3 nM) (Table 1).

It is also important to note that binding affinity may change between *in vitro* and *in vivo* conditions, for example, as a consequence of temperature. Huang and colleagues observed a fourfold decrease in the binding affinities of their mGluR1 tracers when the temperature of the binding assay was raised from 4°C to 37°C [21]. However, for this explanation to apply to ^11^C-LY2428703, the difference between *in vitro* and *in vivo* affinities for mGluR1 would have varied across species. That is, the *in vitro* affinity in humans (about 2 nM) was similar to that in rats (0.6 nM), although PET imaging was successful only in rats.

Another complicating factor is low delivery to brain (i.e., low drug exposure), which may have cause low brain uptake. We compared five different radioligands from previous studies with regard to exposure to brain (Table 3) and measured the area under the curve of plasma concentration vs. time from 0 to 20 min. To determine whether decreased exposure was due to rapid clearance from plasma or low free fraction, we calculated AUC_0 to 20_ for the total concentration of radioligand in plasma as well as a variable we termed ‘effective exposure,’ i.e., *f*_p_ × AUC_0 to 20_. The effective exposure was thus named because only the free concentration of drug can cross the blood–brain barrier. ^11^C-LY2428703 had the highest exposure value among the tracers tested. This high exposure was due to the slow wash-out of the tracer from the vascular compartment and to negligible metabolism in plasma, that is, parent radioligand represented 99% of plasma radioactivity even at the end of the 2-h scanning session. Interestingly, due to its very low *f*_p_ (0.094%), ^11^C-LY2428703 displayed the lowest effective exposure among the five tested ligands. Thus, high binding to plasma proteins (i.e., low values of *f*_P_) markedly decreased effective exposure of the radioligand to brain.

**Table 3:** Brain exposure for five different radioligands

	^**11**^**C-LY2428703**	^**18**^**F-FMPEP**[22]	^**18**^**F-SP203**[19]	^**11**^**C-( *R *)-rolipram**[23]	^**11**^**C-NOP1A**[24]
Target	mGluR1	CB1	mGluR5	PDE4	NOP
Brain peak SUV	~0.5	3 to 4	~6	2 to 2.5	5 to 7
Exposure SUV (0 to 20)	202.2	47.8	37.0	124.9	36.7
*f*_p_ %	0.094	0.63	5.2	6.4	10.1
Effective exposure (*E* × *f*_p_)	0.19	0.30	1.9	8.0	3.7

Finally, with regard to binding potential in the brain, we assumed that equilibrium or near-equilibrium binding conditions existed in the brain at the time of peak brain uptake. Following the Michaelis-Menten equation for receptor binding:

(1) $$B=F\times B_{\max}/\left(K_{D}+F\right)$$

where *B* is the specifically bound ligand and *F* its free concentration. Because *F* <<*K*_D_ in this PET study using tracer doses of radioligand,

(2) $$\begin{array}{ll} B & =F\times \left(B_{\max}/K_{D}\right)=F\times B_{\max}\times \mathrm{affinity}\\ & =F\times \mathrm{BP} \end{array}$$

Following the consensus nomenclature [25], *F* = *C*_FND_. Furthermore, *C*_FND_ is itself linearly related to *f*_p_, as shown below:

(3) $$\text{At equilibrium},C_{\mathrm{FND}}=C_{\mathrm{FP}}$$

where *C*_FND_ is the concentration of the free ligand in the nondisplaceable compartment and *C*_FP_ is the concentration of free ligand in plasma.

(4) $$C_{\mathrm{FND}}=f_{\mathrm{ND}}\times C_{\mathrm{ND}}\;\mathrm{and}\;C_{\mathrm{FP}}=f_{p}\times C_{P}$$

(5) $$C_{\mathrm{FND}}=f_{p}\times C_{P}\;\mathrm{or}\;C_{\mathrm{FND}}\;\alpha \;f_{p}$$

Thus, we were able to calculate effective *BP* (which corrects *BP* for plasma free fraction) as *f*_p_ × *B*_max_/*K*_D_, which equals the specific volume of distribution *V*_S_. Notably, monkeys (0.161) had a tenfold higher effective *BP* than humans (0.016). In fact, although *B*_max_/*K*_D_ for monkeys (18.2) was similar to that for humans (16.9), the *f*_p_ value for monkeys (0.884%) was much higher than that for humans (0.094%). Due to their higher *f*_p_ (2.7%), rats had the highest effective binding potential (0.683) of the three species (Table 1).

The terms effective exposure and effective *BP* help reinforce the fact that *f*_p_ influences not only the amount of radioligand exposed to the brain (and thus available for uptake) but also the maximal amount of drug that the brain can retain at equilibrium. Thus, the single greatest limitation of ^11^C-LY2428703 may be excessive binding to plasma proteins. Indeed, ^11^C-LY2428703 is highly lipophilic: the measured LogD at room temperature in octanol was 4.02 [10]. High lipophilicity is associated with high binding to plasma proteins and high nonspecific binding in brain [21,26]. The two mGluR1 ligands that showed a high signal in the monkey brain, ^18^F-MK1312 [8] and ^18^F-FITM [5], displayed a lower lipophilicity than our tracer (with LogD values of 2.3 and 1.5, respectively).

Although the terms effective exposure and effective binding potential demonstrate the dual roles of plasma protein binding, it is important to note all three factors that affect specific binding in brain: *F*, *B*_max_, and *K*_D_ (Eq. 2). Thus, an unusually low *F* (caused by low *f*_p_) could be compensated for by an unusually large *B*_max_ or by an unusually small *K*_D_ (i.e., high affinity). Such is the case for ^18^F-FMPEP-*d2* binding to the cannabinoid CB1 receptor in brain (Table 3). This highly lipophilic radioligand has an unusually low free fraction in plasma (*f*_p_ = 0.63%). Nevertheless, CB1 receptor density is very high and among the most abundant G-protein coupled receptors in mammalian brain [27]. Thus, ^11^C-LY2428703's unusual property of high binding to plasma proteins creates a reservoir of radioligand in plasma that restricts delivery to and retention in the brain, ultimately impeding its utility as a useful radioligand.

The present study also measured the dosimetry from ^11^C-LY2428703 in monkeys and extrapolated these measurements to humans and obtained a final effective dose of 5.8 μSv/MBq. This figure is well within the range of values for ^11^C-labeled tracers [28]. Indeed, the range of dosimetric results of ^11^C-tracers is uniform enough that we question the utility of dosimetry studies in primates and *a fortiori* in rodents. The radiation safety committee of the NIH similarly endorses this point of view and recently decided that animal dosimetry scans are not necessarily required before first-in-human injections of new ^11^C-labeled tracers. It could further be argued that even human dosimetry is unnecessary at the initial exploratory evaluation of a new tracer. As proposed by van der Aart and colleagues, a standard conservative dosimetry value could be assigned to all new ^11^C-tracers [29]. A full dosimetry study would be performed later only if the initial kinetic evaluation in the brain was favorable.

## Conclusions

Despite very promising *in vitro* and *in vivo* results in rodents, ^11^C-LY2428703 was unsuitable for imaging mGluR1 in monkey or human brain, most likely because of high binding to plasma proteins that restricted delivery to and retention in the brain.

## Competing interests

PZF, SSZ, JSL, RLG, DRF, CM, VWP, and RBI report no competing interests, financial, or otherwise. VNB, EN, ES, and JT are full-time employees of Eli Lilly & Co.

## Authors' contributions

JT and RBI conceived the study and participated in its design and coordination. PZF, JSL, RLG, and DRF participated in the acquisition and analysis of human and monkey data; VNB, EN, and ES performed *in vitro* analyses; SSZ performed blood analyses; and CM and VWP were responsible for the radiochemistry section. All authors read and approved the final manuscript.
